# Effects of different slipping methods on the mortality of sardine, *Sardina pilchardus*, after purse-seine capture off the Portuguese Southern coast (Algarve)

**DOI:** 10.1371/journal.pone.0195433

**Published:** 2018-05-31

**Authors:** Ana Marçalo, Pedro M. Guerreiro, Luís Bentes, Mafalda Rangel, Pedro Monteiro, Frederico Oliveira, Carlos M. L. Afonso, Pedro Pousão-Ferreira, Hugues P. Benoît, Mike Breen, Karim Erzini, Jorge M. S. Gonçalves

**Affiliations:** 1 Centre of Marine Sciences (CCMAR), University of the Algarve, Faro, Portugal; 2 Campus de Excelencia Internacional del Mar (CEI.MAR), Universidad de Cádiz, Plaza de Falla, Cádiz, Spain; 3 IPMA - Portuguese Institute for the Sea and Atmosphere/EPPO - Aquaculture Research Station, Olhão, Portugal; 4 Fisheries and Oceans Canada, Gulf Fisheries Centre, University Avenue, Moncton, New Brunswick, Canada; 5 Institute of Marine Research, Bergen, Norway; Universita degli Studi di Bari Aldo Moro, ITALY

## Abstract

The effects of two different slipping methods on the survival, physical and physiological response of sardines, *Sardina pilchardus*, captured in a purse-seine fishery were investigated in southern Portugal. Sardines were collected and transferred into holding tanks onboard a commercial fishing vessel after being captured, crowded and deliberately released using two slipping procedures: standard and modified. The standard slipping procedure aggregated fish at high densities and made them “roll over” the floatline, while the modified procedure aggregated the fish at moderate densities and enabled them to escape through an opening created by adding weights to the floatline. Both slipping methods were compared with minimally harmed non-slipped sardines (sardines collected from the loose pocket of the purse seine). Survival rates were monitored in captivity over 28 days using three replicates for each treatment. The estimated survival of sardines was 43.6% for the non-slipped fish, 44.7% for the modified slipping and 11.7% for the standard slipping treatments. Scale loss indicated the level of physical impact experienced, with dead fish from the non-slipped and modified slipping technique showing significantly lower scale loss than those fish from the standard slipping treatment within the same period. Of the physiological indicators of stress measured, cortisol, glucose, lactate and osmolality attained peak values during slipping and up to the first hours after introduction to captivity. This work indicates that although delayed mortality after release may be substantial, appropriately modified slipping techniques significantly enhance survival of slipped sardines.

## Introduction

Fish discarding continues to be one of the most important issues in marine fisheries management. It is considered a waste of resources that contributes to overfishing and uncertainty to stock assessments [1–3]. This is mostly due to the potential mortality of fish and other animals that are released or discarded from fishing gears, the magnitude of which is difficult to estimate [4]. Discard quantification has been a priority mostly directed at less selective and more abrasive fishing gears, namely towed (e.g. trawling) or static gears (e.g. gill nets, traps) [5–8]. Major efforts have been made to reduce discards, especially through gear and/or operation modifications during fishing that improve species and size selection [6, 8, 9–13]. The continuing high pressure on marine ecosystems from unsustainable fishing practices and the urgency to reduce discarding led to the reformed European Common Fisheries Policy (CFP), to promote a discard ban through the enforcement of a landing obligation to European fisheries to be fully implemented by 2020 [14]. To accomplish this, European governments are now committed to finding ways to address introducing the “Landing Obligation”. This is recognised as a complex challenge and will need to follow a multi-actor approach, whereby fishers, processors, managers, scientists, technologists and NGOs work collaboratively to provide the scientific and technical basis to achieve the gradual elimination of discards in a case-by-case scenario approach.

The deliberate release of pelagic-schooling fishes at the end of purse-seine fishing operations, also referred to as slipping, is a manoeuvre traditionally used to avoid excess or unwanted catch due to regulatory or market demands [15–16]. Slipping is a process where part of the catch is released by rolling the fish over the headline (floating line) of the net after partially hauling or “drying-up” the net while it is still in the water [15–18]. This process led scientists and managers to the general assumption that the fishery had a low impact on escapees, because the catch never leaves the water before being released. Thus, for most purse-seine fisheries, slipping is not accounted for and fish released in such condition are assumed to survive. However, for the last decade, a growing body of research has demonstrated that mortality of slipped fish for several small pelagic species (e.g. sardines, sardinops, herring, mackerel) may be substantial, and may result in unacceptably high rates of unaccounted or collateral fishing mortality [11, 15, 16, 18, 19–22]. These studies have shown that mortality of slipped fish is directly related with the conditions and interactions that occur within the net, with mortality increasing with increasing crowding densities and time, and scale loss [18, 20, 22]. These stressors mostly occur at later stages in the fishing event, which coincides with the time at which fishermen have sufficient information on which to decide whether to retain or release the catch. Thus, slipping at an earlier phase in the haul, when the crowding densities are lower, could induce far lower levels of mortality. However, this could present the fishers with to the challenge of accurately characterising the catch before making a decision on whether to release or not, although new methods and technologies to address this are currently being developed (see [23] for review).

The Portuguese purse-seine fishery targeting European sardine (*Sardina pilchardus*) (which belongs to the Southern sardine stock in EU Atlantic waters) is responsible for ~50% in biomass of fish catches landed in mainland ports [24]. More recently, in response to the decline of this sardine stock, restrictive fishing measures have been applied in order to ensure the sustainability of the resource [25]. To maintain economic viability, during the period of the “sardine ban”, the purse-seine fishing sector targets other important small pelagic in the Portuguese pelagic system, such as Atlantic chub mackerel (*Scomber colias*) and horse mackerel (*Trachurus trachurus*), forcing fishermen to discard by slipping any sardine mixed in the catch. The magnitude of slipping in the Portuguese purse-seine fishery has been described as high and variable [15]. Sardine stress during fishing has been described [19, 26], where laboratory studies revealed that survival rates of released fish can be affected by a mixture of operational (*e*.*g*. holding time and density), biological (*e*.*g*. size/condition factor), physical (*e*.*g*. scale loss) and environmental (*e*.*g*. water temperature) factors. These factors are responsible for affecting primary and secondary physiological stress responses and behavioural changes, leading to variable and sometimes high mortality rates [20, 27–28].

**Fig 1:**
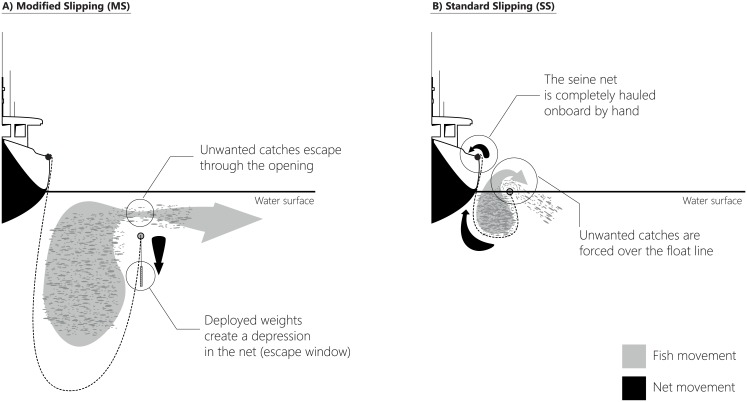
Diagram showing the two slipping methods: a. modified (MS) and b. standard (SS). See main text for detailed descriptions.

The aim of this study was to test methods to minimize the mortality of sardines that were slipped from purse seines capture off the Algarve coast (Portuguese Southern coast). The effects on survival, physiological stress and physical damage of a modified slipping technique (using weights to create an escape window and allow unwanted catch to swim freely out of the net) were compared with those of the standard slipping operation (fish rolled over the headline; Fig 1) and non-slipped and non-crowded sardines, treated here as experimental control subjects. In the experiment, sardines were captured at sea and monitored over a period of 28 days in captivity.

## Materials and methods

### Live capture and transfer of live fish

Live sardines were obtained during a commercial purse seine operation off Eastern Algarve (36° 56´ 33´´ N/ 7° 54´ 53 W) in Southern Portugal (Table 1). The sardines were captured on a fishing trip in late April 2016 at night and in relatively good sea conditions (wave height 0.5–1m). Commercial fishing operations followed the typical purse-seine operation [15, 19] on a day during a “sardine ban”, during which vessels are obliged to release any sardines found in the catch.

**Table 1:** Summary information for the field and monitoring phases with mean values (and ranges) for: Transport density at sea, holding temperature, number of fish, length, weight, condition factor (CF, adapted from Fulton’s “K”) and dead sardines at the end of the study. “Reps” stands for replicates, “Days” stands for days of monitoring.

At Sea								Captivity					
SST (°C)	Depth (m)	Catch (tons)	Species	Treatment	N Reps	N fish	Density (kg/m3)	T (°C)	Length (cm)	Weight (g)	Condition factor	N dead	Days
16.9	39	1.2		Control	3	106	4.2	19.1	14.1	21.7	0.8	55	28
			*Sardina*			(89–126)	(3.5–5.0)	(18.4–22.1)	(13.2–14.9)	(15.7–27.6)	(0.6–0.9)	(47–62)	
			*pilchardus*	Modified slipping	3	118	4.8	19.1	14.0	21.6	0.8	61	
						(108–127)	(4.4–5.1)	(18.4–22.3)	(12.2–15.1)	(16.6–28.3)	(0.7–0.9)	(56–69)	
				Standard slipping	3	143	5,2	19.5	14.0	20.7	0.8	118	
						(134–149)	(4.8–5.5)	(18.4–22.2)	(12.6–14.9)	(15.1–26.8)	(0.6–1.1)	(114–121)	

To obtain sardines for controls (less crowded fish), drying up of the net was interrupted at its final stage to avoid additional damage. To facilitate fish transfer, crew members and researchers collected fish swimming within the netted area and transferred them from the net directly to the transport tanks of the fishing vessel using 10 l buckets and 15 l vinyl scoops. Following this process, the net was bunted (manually hauled) and 4–5 sets of 10 kg weights were put along the headline to form an escape opening (Fig 1a). This operation was called the modified slipping manoeuvre, with sardines freely swimming out of the net through the opening. Next, within 10–15 minutes, drying up was completed and fish were released from the net by “rolling” them over the headline and into the sea (standard slipping; Fig 1b). Samples of escaping fish were collected during each slipping operation, using the same methods as used for the controls. Small numbers of fish were transferred in each operation (five to 20 fish per bucket or scoop) to minimize physical contact among fish and avoid damage through abrasion [20, 27, 29]. Fish transfer was fast (~5–10 min for each treatment) and around 300–400 sardines were collected for each treatment and placed into three 600 l rectangular-blue transport tanks (3 replicates per treatment) previously filled with oxygenated sea water. Fish stocking densities were visually adjusted and deviations from intended transport densities occurred, but in most cases densities <5 kg/m^3^ were achieved (Table 1) as suggested in [27]. After the conclusion of transfer operations, tanks were covered and the aeration system was re-adjusted. Oxygen levels were regularly inspected with a portable oxygen meter (YSI, YSI Incorporated, Ohio, USA) and the flow of the aeration pump was frequently adjusted to maintain saturation levels in the tanks close to 100%. On arrival at the port, which took more or less about 48 minutes, each tank was moved onshore with the help of the vessel crane, and transported by road to the Aquaculture Research Station of IPMA in Olhão (5 min per trip). The total duration of transport and moving the fishes to the aquaculture tanks took around 90 min for the 9 tanks.

### Maintenance in captivity

#### General monitoring

At the Aquaculture Research Station, sardines in each transportation tank were rapidly transferred into 3000 litre outdoor black-circular holding tanks with the help of vinyl—hand nets and buckets (Table 1). An open-system water circulation and variable water flow (minimum of 1.8 m^3^/hour) was used, with an aeration inlet placed at the centre of the tank to facilitate the natural circular movement of schooling fish. Nets were used to cover each tank to avoid fish jumping out. In all cases, fish were kept under a natural light regime and photoperiod. Although refrigeration was activated during the experiment to maintain water temperature in the holding tanks at the station facilities as close as possible to the temperature of the time of capture and transportation, daily variations in water temperature were observed (Table 1). Fish were fed with dried pellets (1–3 mm diameter pellets; Aquasoja-Sorgal, S.A., Ovar, Portugal) at a daily rate of 1–2% biomass (wet mass). Gilthead seabream (*Sparus aurata*) and meagre (*Argyrosomus regius*) eggs when available (during the first two weeks in captivity) at the aquaculture station were also provided and fed to the sardines. Feeding started with a few individuals initially leaving the shoal and exploring the pellets or fish eggs, usually during the first week in captivity. Faecal matter and uneaten food pellets were regularly siphoned from the bottom of the tank and daily water purges of a quarter of the tank were performed to promote water quality by eliminating any excess ammonia from excretions. The monitoring study period lasted 28 days.

#### Survival

The holding tanks were monitored daily, recording water temperature, fish behaviour and the number of deaths. Dead fish were removed from the tank twice daily (in the morning and late afternoon) to maintain good water conditions, placed in individual plastic bags and frozen for subsequent biometrical measures, quantification of scale loss and to tabulate daily mortalities. All were measured for total length (TL) and weighed (total body mass, BMtot). Fish condition (condition factor, CF) was calculated as CF = 100*BMtot/TL^3^, based on Fulton´s “k” factor. Assessment of scale loss was performed by adapting the method of [30], as described in [20, 27] which involved dividing one flank of each fish in eight regions that could be delimited visually, and scale loss in each region evaluated on a level of 0 to 10 (corresponding to 0 to 100% scale loss).

#### Biochemical analysis

Blood sampling was performed on live anesthetised fish individually collected at sea, at station arrival and from the experimental tanks using a hand-net. Blood samples from 10 fish from the three treatments (control, modified slipping and standard slipping) were taken immediately after capture at sea (day -1) to describe early post-capture evolution of physiological variables. Similar blood sampling was also performed immediately upon arrival of the transportation tanks at the IPMA station (day 0) and then on days 2, 7 and 28 (Table 2). Fish from a treatment were collected all at once and immediately placed in a small sedation tank with 2-phenoxyethanol (1:250). Two researchers were responsible to withdraw blood which took about 0.5 min per fish. Once fish were fully sedated (within 1–2 min), blood was rapidly collected from the caudal vein (usually 0.2–0.5 ml) with a heparinized 1-ml syringe fitted with a 23G needle. This handling procedure resulted in sufficient harm that fish were immediately humanely euthanized, by severing the spinal cord behind the head.

**Table 2:** Summary of biological data (mean and standard deviation) for sardines sampled for blood analysis in the three treatments.

		Day	Length (cm)		Weight (g)		Condition factor		N
			Mean	SD	Mean	SD	Mean	SD	
**Control**	**Fishing**	-1	14.2	0.3	24.8	2.0	0.9		10
	**Monitoring**	0	14.1	0.4	21.6	2.6	0.8		10
		2	14.3	0.3	22.7	1.6	0.8		10
		7	13.9	0.4	22.9	3.2	0.8	0.1	8
		28	14.6	0.3	25.3	2.8	0.8	0.1	15
**Modified slipping**	**Fishing**	-1	14.3	0.5	24.4	2.2	0.8		10
	**Monitoring**	0	14.1	0.4	21.2	2.1	0.8		10
		2	14.4	0.5	22.5	2.0	0.8	0.1	10
		7	13.6	0.4	20.7	2.0	0.8	0.1	9
		28	14.3	0.4	25.6	3.8	0.9	0.1	15
**Standard slipping**	**Fishing**	-1	14.1	0.4	23.6	2.1	0.8		10
	**Monitoring**	0	13.8	0.6	22.2	1.3	0.8	0.1	10
		2	14.4	0.5	23.3	2.0	0.8		10
		7	13.9	0.4	22.0	2.2	0.8		6
		28	14.3	0.4	25.5	3.3	0.9	0.1	15
**Post stress**	**“Low stress”**	49	15.1	0.4	31.9	3.6	0.9	0.1	20
	**“High stress”**	49	15.0	0.4	28.1	3.7	0.8	0.1	18

To provide reference to the physiological responses observed in the main experiment, it was necessary to conduct a secondary experiment to establish a range of physiological responses to controlled stressors. Complementary information on low and high levels of cortisol, glucose, lactate and osmolality was obtained from sardines separated in two different groups (“low stress” and “high stress”) sampled at day 49. For this, at the end of the experiment (day 28), remaining survivors were distributed into two separate black-circular holding tanks and kept (regular feeding and tank maintenance took place) for about two weeks with minimum disturbance. At the end of this period (day 49 after capture), 20 sardines from one tank were immediately removed for blood sampling and about 18 sardines from the second tank were subjected to an intensive stress effect, using a combination of air-exposure, chasing and confinement, by lifting them out of the water for 1 minute and then transferred to a small aerated bucket (about 10 l) for about an hour with chasing events every 15 minutes. To avoid additional suffering, these fish were swiftly sedated in the holding bucket with a lethal concentration of 2-phenoxyethanol, after which blood samples were rapidly obtained and then the fish humanely euthanized within 1 minute.

After collection, blood was immediately refrigerated and centrifuged at 5000 g for 3 min and the plasma stored at -20° C for subsequent analysis of osmolality, cortisol, glucose, lactate, plasma protein and plasma lipids at CCMAR- University of Algarve. Plasma samples were defrosted and osmolality was measured in 10 μl samples with a vapour pressure osmometer (Wescor 5520). Plasma cortisol was determined using a radioimmunoassay (RIA) as previously described [31–32]. Briefly, plasma samples were diluted in phosphate buffer containing 0.5 g l^-1^ gelatine, pH 7.6, and heat-denatured at 70ᵒC for 30 min. The antiserum used for the assay was raised in rabbits against cortisol-3-(O-carboxymethyl)oxime–bovine serum albumin conjugate (Sigma-Aldrich). Samples were incubated overnight with fixed amounts of antisera and tritiated cortisol, [1,2,6,7-^3^H(Hydrocortisone)] (PerkinElmer). Bound and free phases were separated with charcoal and the remaining beta-activity measured with scintillation cocktail (Ultima Gold, PerkinElmer) in a Microbeta Trilux Detector (PerkinElmer). Plasma glucose and lactate were measured by enzymatic colorimetric methods using commercial kits from Spinreact (Glucose-Ref. 1001190; Lactate Ref. 1001330) adapted to 96-well microplates. Plasma total lipids were determined with the sulfo-phospho vainilline colorimetric assay using the Spinreact kit (Ref. 1001270) adapted to 96-well microplates and total protein was measured with the Quick Start^™^ Bradford Protein Assay (Biorad Cat. # 500–0202). All colorimetric measurements were performed with a Benchmark Microplate Reader (Biorad).

### Ethics statement

All experimental procedures were performed in accordance with the Guidelines of the European Union Council (86/609/EU) and Portuguese legislation for the use of laboratory animals, under licences (Permit numbers 0421/000/000/2013 and 010238 from 19/04/2016) from the Veterinary Medicines Directorate (DGAV), the Portuguese competent authority for the protection of animals, Ministry of Agriculture, Rural Development and Fisheries, Portugal. The rules and regulations which protect experimental animals from unnecessary pain and suffering have been strictly followed during the experiment and the experiment design considered the application of the 3R (Replacement, Reduction and Refinement) policy and the 2010/63/UE directive. Fish were obtained during commercial operations and maintained at the state-of-the-art fish culture facilities (EPPO) of the Portuguese National Institute for Sea Research (IPMA). In captivity, individuals used in this study were kept under optimal density, water and feeding conditions. From a total of 1102 sardines, 196 (17.8% of total) were used in samplings and sacrificed (i.e. the minimum number to carry out statistical analysis), with a lethal dose of anaesthesia (1:250, 2-phenoxyethanol) followed by euthanasia by cervical section. All activities were conducted by or under the supervision of researchers trained on animal experimentation and welfare and duly licensed by DGAV. This study was designed to take advantage of a commercial fishing operation to evaluate several methods to increase survival of discarded (released) fish. This is a process which always leads to immediate and delayed mortality. Mortality rates in these operations are unknown and estimated to be highly variable. Fish discarded during the fishing procedure were collected and maintained in optimal controlled conditions. Any mortality was derived from the commercial fishing process. Since the aim was to estimate survival/mortality during time after fishing, it was not possible to apply humane endpoints. All other fish sacrificed for sampling or analyses were euthanized as described above. The sacrifice of these sardines was crucial to understand factors leading to delayed mortality of discarded fish, thus contributing to finding improved operational techniques that can promote survival of millions of escapees after purse seine fishing.

### Data analysis

For analysis, sampling during capture was considered time (day) -1 and sampling at arrival in captivity named time (day) 0. The survival of sardines over time was modelled using a parametric Weibull mixture distribution model that has previously been applied for discard mortality data [33–34]. This model has been shown to be well suited to this type of data as it models survivorship as a decreasing function of time for an initial period post release, followed by an asymptote at which the mortality associated with capture and handling is assumed to have been fully expressed. The model is fit to time of mortality for individual fish and can accommodate censored observations as in the present case, right censored observations for fish that were removed alive from the experiment when it was terminated at 28 days and for which the time at mortality is known only to occur after the time of censoring.

The Weibull mixture-distribution model is based on the assumption that the sample of fish in a given treatment is composed of two groups, one comprising individuals that were adversely affected by capture and handling and which will die as a result, and one comprising individuals that were not adversely affected and that will survive the period of captivity. The model can be written as follows (see [33] for a derivation):

$$P(T>t)=S(t)=1-\pi +\pi S_{A}(t)$$ (1)

$$S_{A}(t)=exp(-({\alpha t)}^{\gamma })$$ (2)

Where *T* is the time of death, *π* is the probability that a fish was adversely affected by capture and handling, *S*_*A*_(*t*) is the “short-term” survival function for the affected group, and *α*>0 and *γ*>0 are respectively the scale and shape parameters of the Weibull distribution. As can be seen in the equations, as *t* tends to infinity, *S*_*A*_(*t*) tends to zero and the overall survival function *S*(*t*) becomes 1- *π*, the long term post-release survival rate. Model parameters were estimated by maximisation of the model likelihood, and model fit was assessed visually by comparing the estimated model survival function and non-parametric Kaplan-Meier (KM) curves for the data, which express the proportion alive as a function of time, while accounting for censoring in the data [35].

The model was first fit independently to the data for each treatment and replicate to compare parameter estimates between them. A small suite of candidate models that were fit to the ensemble of data from the experiments were then compared. The first model (M1) assumed that the survival functions did not differ between treatments, that is that single common values for each of the model parameters (*α*, *γ*, *π*) could explain survivorship over time for all treatments. The second model (M2) assumed that the long term post-release survival rate differed between treatments by incorporating a treatment-factor effect on *π*, but assuming that the values for the two other parameters were common to all treatments (see [34] for details). The third model (M3) assumed that both the long term post-release survival rate and the Weibull rate *α* differed between treatments. This means that both the survival asymptote and the rate at which it is reached could vary between treatments. Finally, the fourth model (M4) assumed that all three parameters of the Weibull mixture model could vary between treatments. The relative evidence for the models was assessed using Aikaike’s Information Criterion corrected for small sample size (AICc).

By rearranging Eqs 1 and 2 and solving for *t*, it is possible to estimate the time at which the survival asymptote is essentially reached (ie. when 99.9% of individuals have died) as

tasy=1αln(103)1γ (3)

This is a useful parameter to estimate as it can be used to confirm that the period of captivity was long enough to capture all mortality associated with capture and handling, including delayed mortality. It can also be used in planning future captivity studies. In addition to estimating *t*_*asy*_, a small simulation exercise was also undertaken using the data collected in the study to determine the sensitivity of survival asymptote estimates for sardines to the duration of captive observations. For each interaction, the maximum study duration was progressively truncated by one day. The observation time for all censored observations was set to the truncated time and fish that had been observed to die after that time were changed to censored observations. The mixture-distribution model was then fit to the truncated data to obtain an estimate of the survival asymptote. This procedure replicated the data and model parameter estimates that would have been obtained had the study been of shorter duration.

The evolution of scale loss (response variable) over time (explanatory variable) and comparison between fish that were dead or alive (fish sampled for biochemistry) at each sampling day was modelled with a generalized linear model (GLM) with an overdispersed Poisson distribution and a log link as described in [27] to explore the association between scale loss and delayed mortality. The GLM model was fitted in R software (R Development Core Team, 2017) and model adequacy was visually inspected through residual plots. Medians of scale loss for all sardines that died during capture (day-1) up to the first 2 days in captivity were compared between treatment groups using a Kruskal-Wallis test followed by pair-wise multiple comparison among groups using Dunn´s method. The change of each blood parameter/variable during capture (day -1) and monitoring in captivity (up to day 28) was analysed separately. Data for each physiological variable were analysed with two-way ANOVA, using treatment and day as independent factors, followed by Tukey HSD test for pair-wise comparisons. The evolution of each blood parameter was also estimated through boxplots plotted using the *ggplot2* package [36]. Low stress and high stress groups were compared by a one-way ANOVA for all physiological variables.

## Results

In the sampled purse-seine catch, 1.2 tons of sardine (almost clean catch with >95% sardines and ~5% chub mackerel) were caught and 1102 sardines (23.5 kg) were transferred alive to the transport tanks onboard the purse seiner. There was little between-treatment variation in sardine size (mean TL = 14.0 cm; mean BMtot = 21.3 g; Table 1). Sea and land transportation mortality was low for control (C) and modified slipping (MS) tanks (respectively 4.7% and 3.9%), and moderate in standard slipping (SS) tanks (16.6%). Fish transportation density differences for each treatment occurred, due to the fast transfer operation of the fish from the net to the transportation tanks and uncontrolled visual estimation support. However, these differences were small, particularly between the MS and SS treatments and are unlikely to have pronounced effect on sardine mortality (Table 1). Consistent and considerable weight (BMtot) and condition factor (CF) gains were observed for all surviving fish in captivity at the end of the observation period in all treatments (Table 2).

### Survival

**Fig 2:**
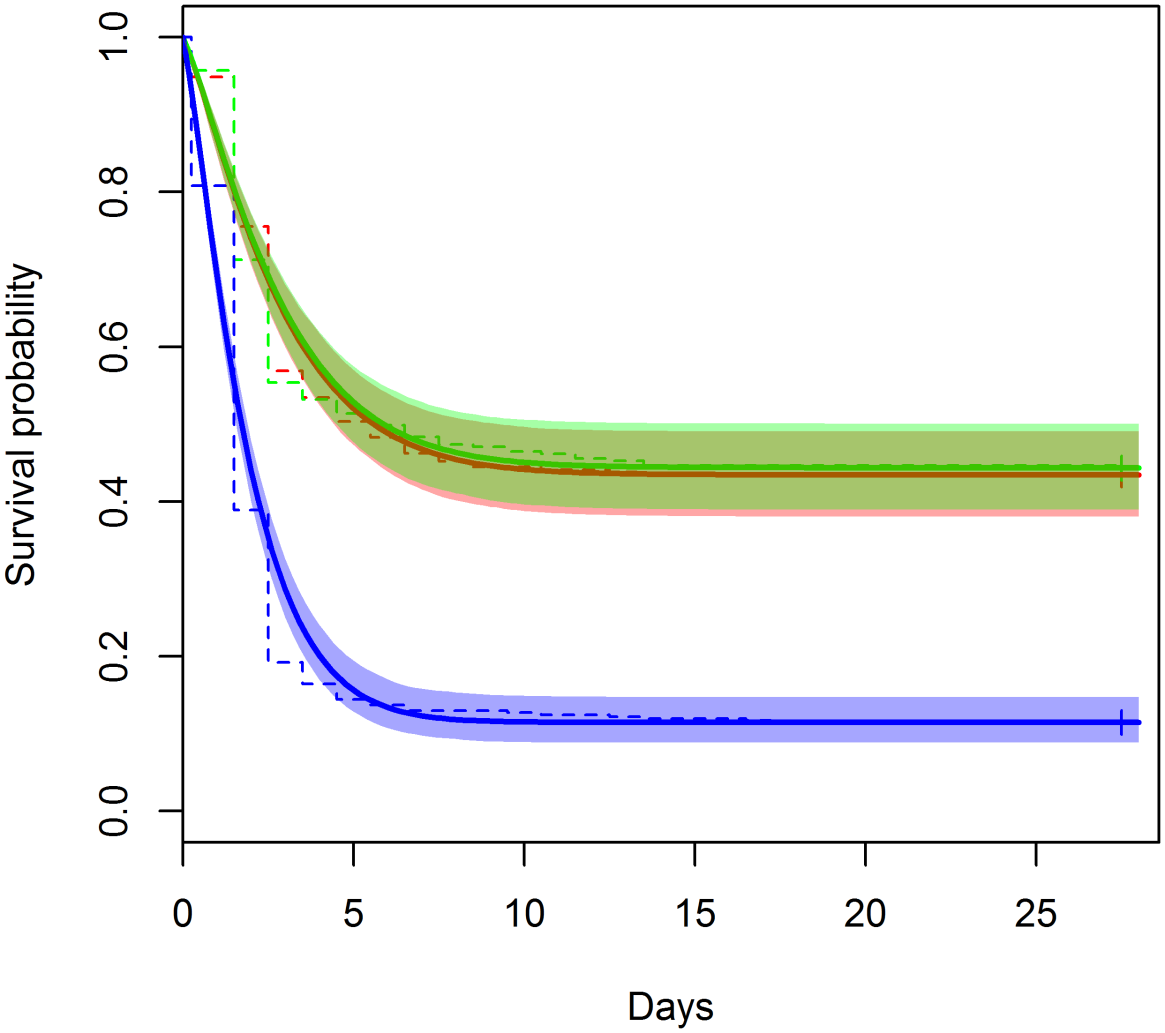
Survival estimates of sardines by Kaplan-Meier survival estimator (dashed line). Overlay with the predictions from the selected mixture-distribution survival model M3 (solid lines with 95% confidence bands) for the three treatments: Control (Red), Standard slipping (SS-Blue) and Modified slipping (MS-Green).

Sardine survival for each treatment as a function of observation days is shown in Fig 2. Patterns indicated that mortality had reached asymptote early in the observation period. In all treatments higher mortality occurred within the first 3–5 days, followed by low rates of mortality in the remaining period in captivity, confirming prior observations made after commercial or simulated fishing [20, 27–28]. At the end of the monitoring period, a total of 701 sardines had died (164 in Control, 182 in MS and 355 in SS; Table 1). For all treatments, small between-replicate variation on survival rates was observed (S1 Fig).

**Fig 3:**
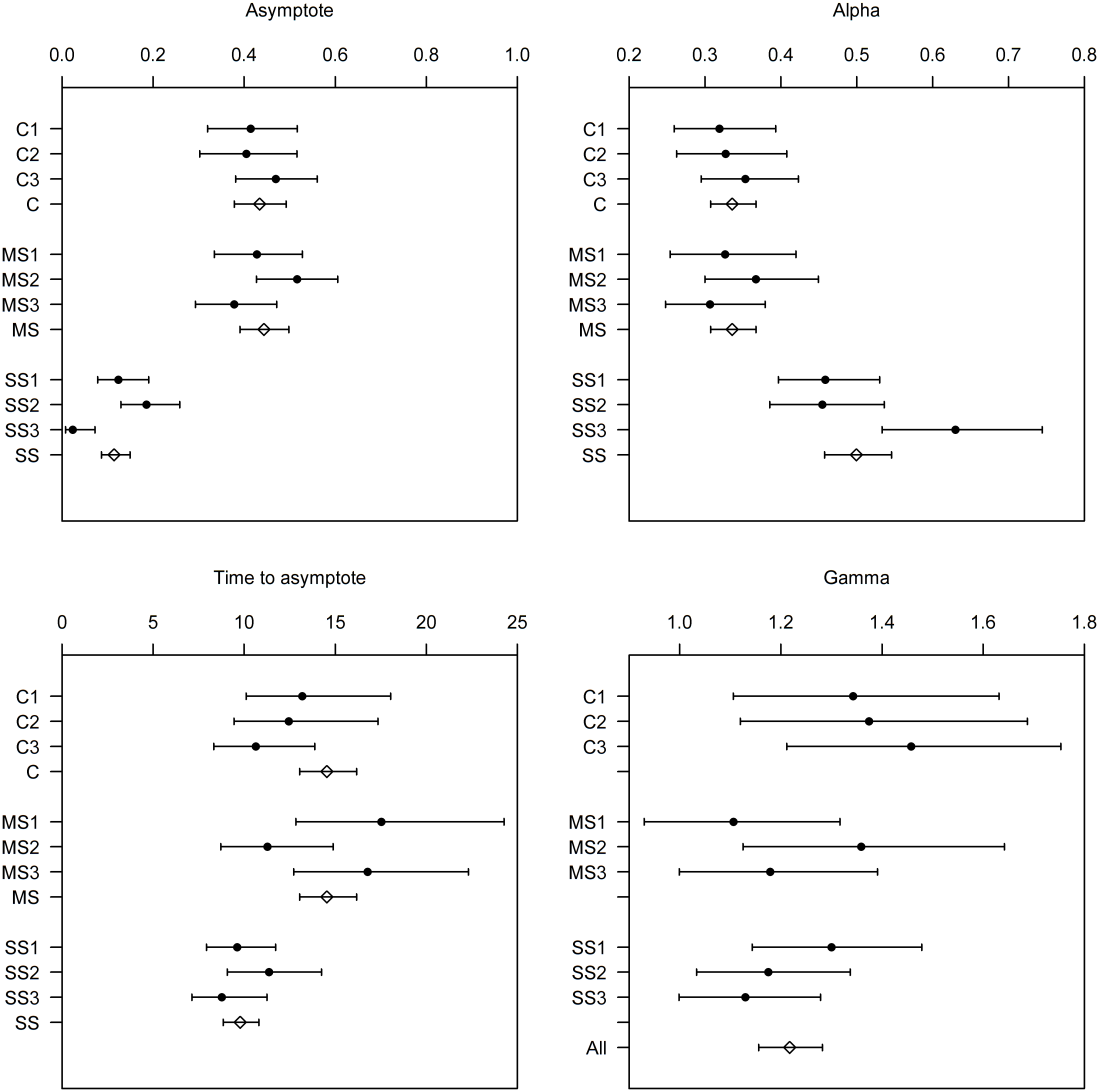
Estimated model parameters for control (C), modified slipping (MS) and standard slipping (SS) treatments. Numbers indicating individual replicates and the absence of numbers indicating values from the selected candidate model fit to data pooled across replicates. The dots are the maximum-likelihood estimates and the bars are 95% confidence intervals. The individual panels are for a) alpha and b) gamma, respectively the rate and shape parameters of the Weibull distribution, as well as c) the estimated survival rate at the asymptote and d) the time required to reach the asymptote (day).

The four candidate models provided different fits to the aggregated data based on AICc values: M1–3944.0, M2–3817.2, M3–3777.1, M4–3780.9. From these results the preferred model was M3, which assumed that both the long term post-release survival rate and the Weibull rate *α* differed between treatments. This model fit the data well (Fig 2; see parameter estimates in Fig 3, open symbols). Survival at asymptote (with 95% CI) was estimated at 43.6% (38.0, 49.3) for the C, 44.7% (39.3, 50.1) for the MS and 11.7% (8.9, 15.2) for the SS (Fig 3c) treatments. The estimated time to asymptote was shorter for the SS treatment at 9.8 days (8.9,13.0) compared to the other two treatments 14.6 days (10.8, 16.2) (Fig 3d), but all were well within the study duration of 28 days confirming that all delayed mortality had been observed. The Weibull mixture-distribution survival model provided a good fit to individual replicates (S1 Fig). Although there was a slight tendency for the model to underestimate the rate of decline between days 1–4, the survival at the asymptote was very well estimated. The estimates of model parameters were consistent among replicates within treatments and were very similar for the C and MS treatments, but different for the SS treatment (Fig 3). The alpha (*α*) parameter was higher for the SS treatment, meaning that the individuals that were adversely affected by capture and handling died at a faster rate, which resulted in a slightly more rapid time to asymptote (*t*_*asy*_). The gamma (*γ*) parameter did not vary markedly between treatments.

**Fig 4:**
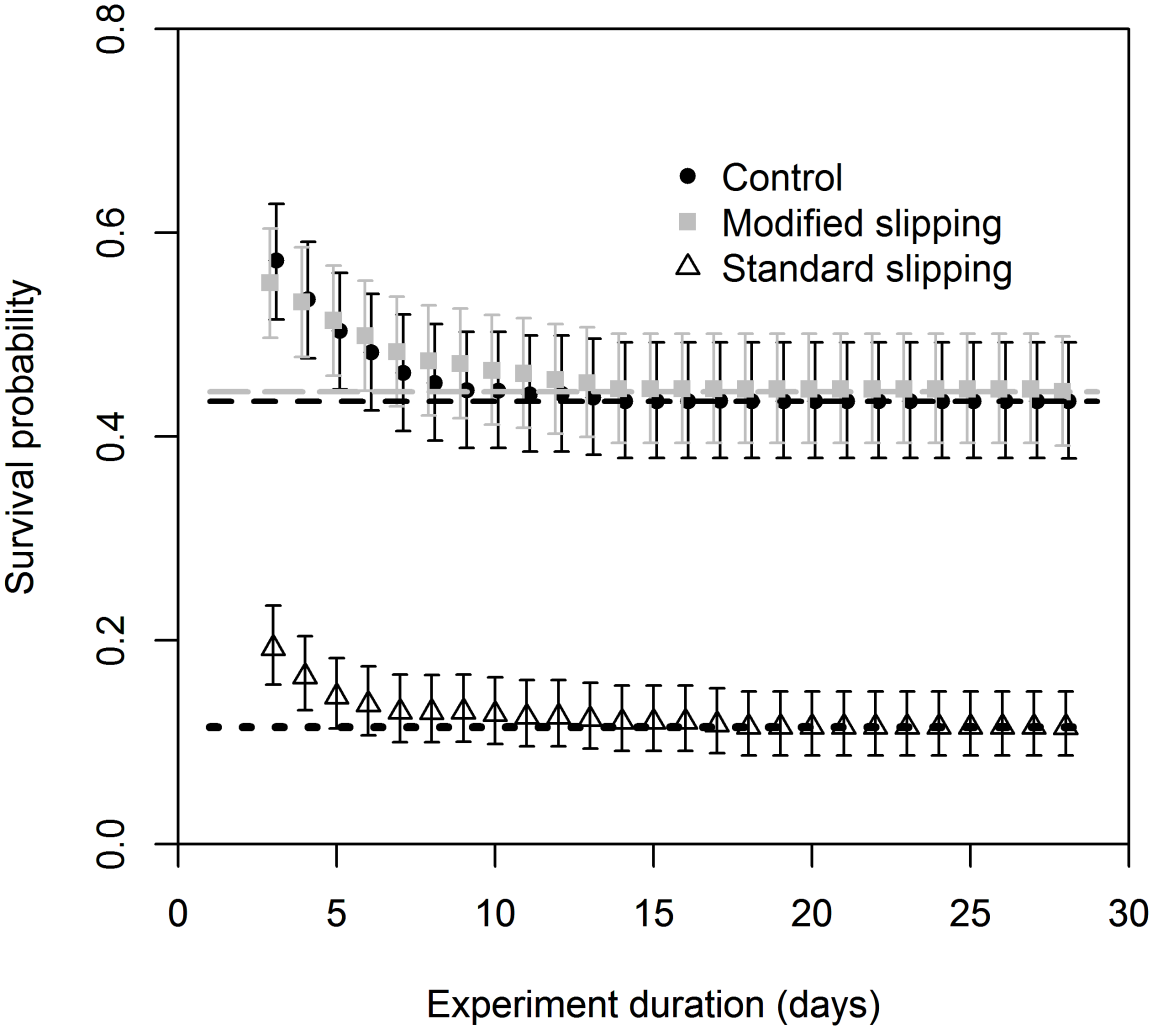
Effect of study duration on the accuracy of the estimated survival rates at asymptote for the control (black circles), MS (grey squares) and SS (open triangles) treatments. The symbols are the maximum-likelihood estimates and the bars are the 95% confidence intervals for the estimated survival rate at asymptote. Lines indicate the survival asymptote estimates for each respective treatment based on the original data.

The simulation for study duration indicated that the captive duration in the study could have been as short as 7–8 days, with little consequence for biased estimation of the survival at the asymptote (Fig 4). As study duration is reduced below 7 days the bias increases rapidly with underestimation of the survival rate.

### Physical injury

**Fig 5:**
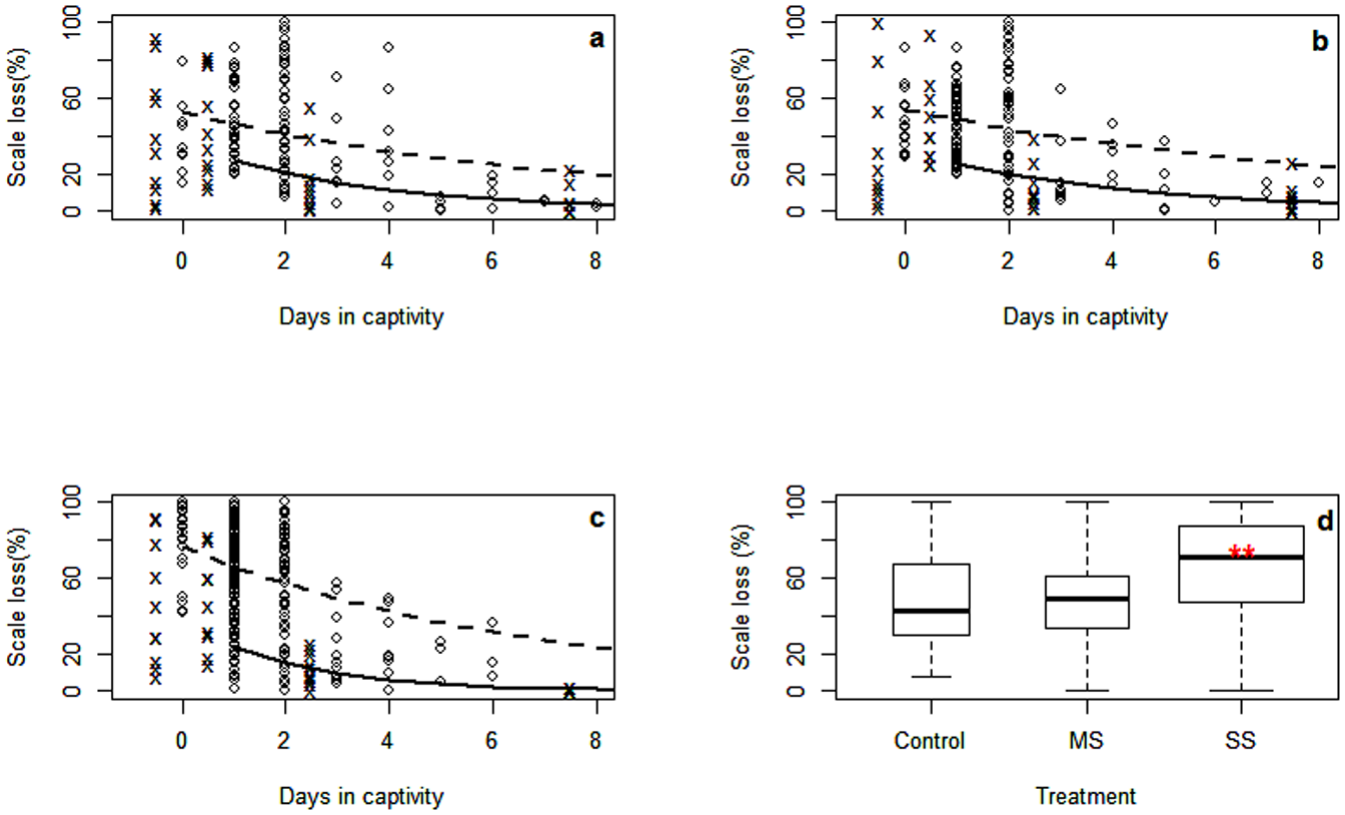
Sardine scale loss (%) during the first week in captivity, separately for fish sampled dead (O) and alive (x) in treatments a. control, b. modified slipping and c. standard slipping. Sampling day for live fish is shifted by 0.5 days to improve clarity. Lines indicate fitted GLM (shown in Table 3; solid line, live fish; dashed line, dead fish). d. Distribution (boxplots) of scale loss for all sardines that died during capture (day-1) up to the first 2 days in captivity in the three sampled treatments (0-control; 1—modified slipping; 2—standard slipping). ** symbol indicates significance of SS treatment over the other two treatments (Kruskal-Wallis: *H* = 73.3, d.f. = 2, *P*<0.001).

For all treatments, physical damage was associated with the probability of dying, with dead fish having significantly higher level of scale loss (p< 0.001; Table 3; Fig 5a–5c). In the models for all treatments significant differences in the intercept were found between dead and alive fish. The difference in slope was significant (p< 0.05) for the C and MS treatments and very significant (p< 0.001) for the SS treatment. Further, as observed previously in [27], we cannot underestimate the fact that scale loss through abrasion certainly might not only have occurred during the operations at sea, but could have been prolonged until the introduction of fish to the holding tank. However, from then onwards, once more, the results show that fish with most severe scale losses die first for both groups of fish analyzed (dead and live). Consistent reduction of mean scale loss with time in captivity was significantly lower in the SS treatment compared with the C and MS (Table 3). Median sardine scale loss for the first two days in captivity for the slipping treatments was 48.8 and 70.6% for the MS and SS respectively, with the SS treatment showing significant differences from the C (42.3%) and MS treatment (Kruskal-Wallis: *H* = 73.3, d.f. = 2, *P*<0.001; Dunn´s test: P>0.05 for the relevant pair groups; Fig 5d).

**Table 3:** Summary statistics of GLM fitted to fish scale loss as a function of time in captivity (days) and state (dead or alive) for each sampled treatment (control; modified slipping; standard slipping). The exponent of the intercept corresponds to the mean percentage of scale loss on the first day in captivity (Day 0), while the slope indicates the mean scale loss (percentage). Standard error (S.E.) given in parentheses.

Treatment	Explained	Fish state	Intercept	S.E.	Slope	S.E.
	deviance (%)					
**Control**	15.2	Dead	3.967	(0.076)	-0.126	(0.032)
		Alive	3.560	(0.142)	-0.151	(0.074)
**Modified Slipping**	11.9	Dead	3.987	(0.053)	-0.102	(0.021)
		Alive	3.474	(0.124)	-0.136	(0.057)
**Standard slipping**	10.9	Dead	4.339	(0.034)	-0.149	(0.020)
		Alive	3.574	(0.107)	-0.290	(0.085)

### Biochemical analysis

#### Post capture effects

**Fig 6:**
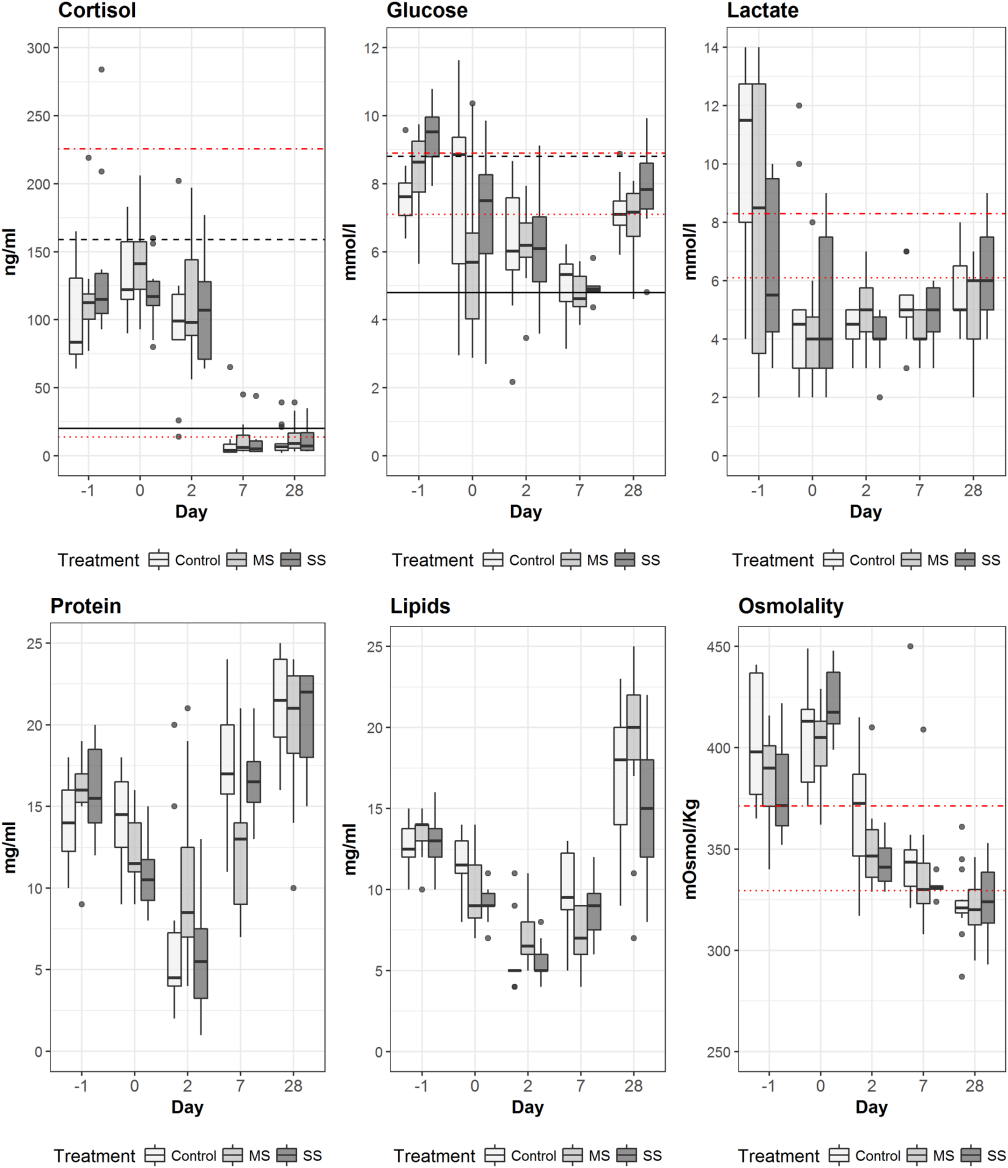
Evolution of physiological parameters in sardine blood plasma for each treatment (C – control; MS- modified slipping; SS – standard slipping) during the monitoring period are shown. Black lines for cortisol and glucose are the mean for observations at the beginning (solid) and at the end of purse seine fishing operations (dashed) calculated from Marçalo et al. (2006); Red lines are the mean of observations for sardines sampled at day 49 for “low stress” (dotted) and “high stress” (dotdash).

Development and comparisons between treatments for physiology parameters are presented in Table 4 and Fig 6. A significant day effect was observed for all variables and a significant treatment effect was observed for osmolality only (ANOVA: Table 4). Cortisol concentration in the plasma was highest at capture and until 48 hours after the introduction into the holding tanks (Day 2) for all treatments, with peak values between 100 and 200 ng·ml^-1^, and decreasing thereafter close to baseline levels (2–20 ng·ml^-1^) from day 7 onwards. Multiple comparisons revealed that significant differences (Tukey, *p*<0.05) were only observed between the C and the SS treatments at sea (Day -1). Glucose levels showed an initial increase for all treatments, which was significantly higher for the SS treatment (*p*<0.05) than the C treatment in fish sampled upon capture (day -1; 9.4±0.3 vs 7.6±0.3 mmol·l^-1^ respectively; Tukey, *p* <0.05). Glucose kept rising in the C treatment up to introduction into captivity and a rapid decrease was observed in fish for the MS and SS treatments during the same period. Glucose levels decreased and were identical in all treatments, and similar to unstressed levels at day 7 (below 5 mmol·l^-1^ in all groups), after which an increase was once more observed, which may have been caused by food intake in captivity.

**Table 4:** ANOVA values for treatments affecting blood parameters analyzed (cortisol, glucose, lactose, protein, lipids and osmolality) at sea (day -1) and during 28 days monitoring in captivity.

Parameter compared	df	SS	MS	*F*	*P*
**Cortisol**					
Treatment	2	20.9	10.5	0.9	0.390
Day	4	4320.1	1080.0	97.9	**<0.001**
Treatment x day	8	85.6	10.7	1.0	0.462
**Glucose**					
Treatment	2	3032.9	1516.4	1.9	0.154
Day	4	62433.8	15608.4	19.5	**<0.001**
Treatment x day	8	9506.5	1188.3	1.5	0.167
**Lactose**					
Treatment	2	1.7	0.4	5.8	0.116
Day	4	8.5	2.1	28.8	**<0.001**
Treatment x day	8	3.4	0.2	2.9	0.064
**Protein**					
Treatment	2	17.9	8.9	0.7	0.521
Day	4	3497.2	8743	63.9	**<0.001**
Treatment x day	8	313.8	39.2	2.9	**<0.05**
**Lipids**					
Treatment	2	40.3	20.2	2.6	0.080
Day	4	2586.9	646.7	82.3	**<0.001**
Treatment x day	8	156.9	19.6	2.5	**<0.05**

Lactate levels were highest at time of capture (peak values above 10 mmol·l^-1^) with significant differences (Tukey, *p*<0.001) observed between the C and SS treatments, despite the substantial individual variability recorded among all treatments. From introduction in captivity up to the end of the monitoring, lactate levels were similarly low for all treatments (about 4 mmol·l^-1^). Plasma protein and lipids levels show similar patterns for all treatments. Initial elevated values decrease up to day 2 in captivity, and increase thereafter. Osmolality was high in all fish collected at sea and even increased slightly during transport until introduction to tanks, but decreased after two days in captivity, decreasing from ca 400 mOsmol·kg^-1^ to 323±16.6, 321±13.3 and 323±19.6 mOsmol·kg^-1^ in resting fish from treatments C, MS and SS respectively. Plasma osmolality was significantly higher for the C treatment compared with the SS (Tukey, *p*<0.05) at day -1 only, resembling the profile of lactate for this same sampling time.

#### Physiological responses to controlled stressors

For all physiological variables sampled for “low stress” and “high stress” levels (cortisol, glucose, lactate and osmolality), there was a significant difference observed in the mean values among treatments. The “low stress” and “high stress” fish treatments had a mean cortisol value of 13.8 ng ml^-1^ and 225.7 ng ml^-1^ respectively. This range covers well our data from sardines sampled at sea. Baseline levels for cortisol were attained for all treatments after a week in captivity. Similarly for glucose, mean value concentrations found in [19] at the onset of fishing at sea and at the final stages of fishing were 4.8 mmol l^-1^ and 8.8 mmol l^-1^ respectively, while results obtained here, at day 49, were respectively for the “low stress” and “high stress” treatments of 7.1 mmol l^-1^ and 8.9 mmol l^-1^. Results in our fishing study reveal that at the final stages at sea (day -1) glucose values were high for all the tested treatments, with sardines in the SS treatment showing the highest values and the only treatment with significant differences from the C (see above). Further, results here show that low levels are attained for all treatments within 7 days in captivity, and very similar to baseline levels (4.8 mmol l^-1^) observed by [19], much lower than the lowest value from the “low stress” treatment sampled in captivity (7.1 mmol l^-1^), where this high value may be related to the novel nutritional status. Lactate and osmolality plasma levels for the species were measured here for the first time. The range recorded in the “low stress”/”high stress” experiment was quite narrow for lactate, with the “low stress” and the “high stress” treatments having a mean value of 6.1 mmol l^-1^ and 8.3 mmol l^-1^ respectively. Again, as for glucose, the lactate levels in the “low stress” treatment were higher than the lowest measured after fishing, and in this case, the levels in fish from the “high stress” treatment were also lower than those sampled at sea. For osmolality, the “low stress” mean value was of 329.4 mOsmol kg^-1^ and the “high stress” treatment of 371.2 mOsmol kg ^-1^.

## Discussion

The results of the present study demonstrate that using a modified slipping technique during purse-seine operations can significantly improve survival of released sardines, with minimal disturbance of fishing operations and potentiate the improvement of on site resource management by fishermen. Commercial purse-seining operations typically end with complete drying up and slipping, which constitutes a stressful event, leading to physiological, physical and behavioural changes, resulting in variable and sometimes elevated delayed mortality of escapees [19–20, 28]. The experiment in this study showed that mortality and scale loss in sardines slipped using the standard method after fishing operations was significantly higher than that observed in the control (not bunted) and modified slipping groups. The mortality estimates among replicates were consistent, which considerably increases confidence in the results. We therefore conclude that the modified slipping method is a promising technique to reduce substantial and unaccounted mortality, most probable due in part to lower levels of physical damage (scale loss) after crowding compared to standard slipping [18, 20, 27, 37].

Sardine survival, mortality rate (as indicated by the slope rate) and corresponding time to asymptote indicate a direct correlation with levels of physical damage (scale loss). Initial mortality, particularly for controls, was unexpectedly high when compared with values obtained by [27], where, sardines were also obtained from a non-bunted net. This difference in results may be due to sardine size related mortality. For sardines [20] and herring [37–38], size is known to influence the survival rate following escape from fishing nets. This is caused by the higher susceptibility of smaller fish to be injured from the physical trauma associated with handling during fishing [9, 39–40]. In our experiment and compared to previous studies, the sardine size averaged 14.0 cm for all treatments, compared to 18.0 cm in [27] or 18.7 cm in [20]. Nonetheless, the benefits of the modified slipping method (MS) seem to be valid as MS survival rates are comparable to the control and three times higher than the survival observed in the standard slipping treatment (SS). As mortality stabilized and sardines gained weight and condition factor within the first 7–8 days in captivity, we can assume that captivity conditions were suitable for all treatments. The same pattern was also observed in captive observation studies on other small pelagic species [20–21 27, 37].

The majority of deaths occurred in the first week with mortality stabilizing in all groups before the end of the observation period, suggesting that this could be reduced. Furthermore, simulations using the Weibull’s mixture model, demonstrated that the monitoring period could have been as short as 7–8 days, with little consequence for biased estimation of the survival at the asymptote. Therefore, to optimise operational costs and minimise any prolonged suffering of the captive animals, future captivity based survival assessments for this species after purse seine fishing, should be planned to have this shorter monitoring period.

Scale loss or skin damage has consistently been associated with fish mortality in previous studies for sardines [20, 27], herring and mackerel [18, 37] escaping purse-seine fisheries. Furthermore, scale loss is linked with post-fishing stress [37, 41–44], which in this work is indicated by increased plasma cortisol and the initial glucose mobilization (hyperglycemia) at the end of fishing, being indicators of well known studied acute fish stress [19–20, 45–49]. For both plasma cortisol and glucose the direction of change agreed with trends overtime observed in the field [19] and in captivity [20, 27]. The increase in cortisol response for all treatments at the end of fishing was significantly higher compared to established limits at the beginning of fishing (20 ng ml ^-1^; [19]) or for “low stress” sardines (13.8 ng ml ^-1^; this study), demonstrating the physiological impact of the fishing process. However, when comparing to the observations in [19], with mean cortisol value concentrations of 20 ng ml^-1^at the onset of fishing at sea and 159 ng ml^-1^ at the final stages of fishing, the stress experienced by sardines in all the three treatments in this study appears to have been somewhat milder, but with similar trend and pattern. Concurrently, glucose was significantly higher in the fish slipped in the standard method compared with individuals sampled in the control and modified slipping treatments, which is indicative of higher energy demands, which was also observed in herring suffering from substantial loss of scales [37].

Moreover, scale loss and associated injury to the integument, has direct effects on skin homeostasis [50], and increases susceptibility to wounds and ensuing infections, further potentiated by the immediate immunosuppressing effects of stress and increased cortisol levels [51–52]. Skin injury may also lead to osmoregulatory impairment associated with epithelial exposure and increased permeability to seawater with changes in blood ion concentration (Na^+^, K^+^, Cl^-^) indicating increased leakage of both ions and water. Stress(ors) alone can disturb the permeability of surface epithelia [46], and adequate cortisol levels, a factor which can promote Na+ and Cl- uptake, are required for maintaining gill and intestine membrane integrity [46, 53]. Elevated chloride concentrations have been previously shown in small pelagic fish in similar conditions [20, 37]. Our osmolality measurements, which reflect the sum of ions and solutes in plasma, provide evidence of significant osmoregulatory disruption, with elevated values at the end of fishing and transport only (Day -1 and 0) that were not observed later. The mechanism that leads to osmoregulatory distress is not clear, but evidence of scale loss associated with increase of plasma ions over a period of days or even water loss across the gills, which alters the internal milieu homeostasis, are usually highlighted as indirect causes of mortality in many fish species [37, 43, 49]. However, it is important to note that, in addition to ion imbalance, the increase in osmolality may be due in part to the accumulation of energetic substrates involved in the stress response.

Sardine lactate was high during the fishing process but at lower levels for both slipping treatments compared to controls at the end of fishing, although a significant difference was only observed when comparing standard slipping to controls. However, lower lactate levels as stress increases may indicate that the highest levels of lactate for the slipping treatments were obtained prior to the sampling time, while the controls, which were less stressed, still showed peak lactate levels (keeping in mind that there was an at least 10 minute gap between treatments, from controls to standard slipped fish). The rapid decrease in lactate appears contrary to most existing literature, where lactate usually rises during exposure to a stressor as a consequence of increased activity under anaerobic conditions, in which glycogen stores are depleted and lactate accumulates in muscle [54] and drains into the bloodstream. This post stress low lactate pattern was already seen in teleosts after capture and acclimation [55], and may indicate that during a recovery period, with less swimming or fighting activity (e.g. period in the transportation tanks at sea), lactate is reconverted to glucose in a gluconeogenesis process [53]. Accordingly, lactate levels seemed to decrease up to 24 hours and stabilize on the days that followed for all treatments.

Similarly, high levels of plasma protein and lipids during acute stress are indicative of protein turnover and increased lipolysis. These processes may constitute a secondary form of energy mobilization, with amino acids and fatty acids being directly oxidized [53]. In our case, it is not clear whether these two parameters were involved in the stress response. Although protein and lipids were higher at sea, they are also elevated at the end of the captivity period and did not show any significant difference within treatments at any of the sampled times. It is possible then that the lower values observed between days 0 and 7 reflect a depletion of internal stores while fish resume normal feeding. On day 3 in captivity, sardines started feeding, showing a gradual increase in feeding activity for both types of feed (fish eggs and pellets) from this day onward. This increase may likely be related to the onset of fish feeding while in captivity, a suggestion which is supported by the individual variability within treatments and the significant differences observed between treatments from day 2 onwards, with correspondence in the changes in weight and condition factor patterns presented in Table 2. As a result, protein and lipid levels show a pattern that correlates positively with weight and condition factor gains, and the high levels of protein, lipids and glucose may indicate an enriched diet, possibly not ideally suitable to sardine metabolism.

Overall, the physiological plasma blood parameters reflect a noticeable correlation with time inside the net at the end of fishing operations only. This suggests that the responses associated with the fishing process may be enough to mask most physiological differences induced by the different slipping processes. However, since the resulting mortality is different between different treatments, one has to conclude that physiological differences must occur, although the physiological data on the days that followed do not seem to correlate with the probability of survival observed for each treatment. Levels of primary physiological stress reactions, as measured by several blood variables, have previously been shown to not be directly associated with observed mortality [20]. However, the acute mobilization of energy substrates and the long term suppressive effects of cortisol on immune functions must, at least partially, modulate the probability of sardine survival after the different fishing procedures, especially at sea, through behaviour alterations that will affect feeding and/or escape from predators, already seen in [28].

## Study limitations

A principal weakness of this study is that all the study subjects were collected from a single fishing event due to logistical and financial constraints. While the replicates constitute pseudo-replicates that under-represent the variability that would almost certainly exist among fishing events, we feel that effect sizes associated with each treatment generally reflect those that would otherwise have been observed with more replication. The operation of the net, from an unpursed state, to moderate pursing that allowed for slipping over the headline, to additional pursing and then release was accomplished in the sequence and timeline that would occur in the fishery. Furthermore the results for survival and scale loss correspond with our expectations. We therefore feel that the results support the conclusion that modified slipping can improve survival compared to standard slipping. However, additional study with replication is required to properly estimate the survival rates associated with each method.

## Conclusions

The results show that survival rates of sardines released upon a commercial fishing process can be improved using not so abrasive slipping techniques. The most important outcome is that complete drying up and ultimately slipping over the headline, causes more physical injury and reduces the probability of survival of slipped fish. It is important to note that the modified slipping technique was suggested by the skipper of the vessel where this work was conducted. In particular, it was claimed that the procedure was commonly used by this skipper to discard unwanted small pelagic species, with the rationale that not only was it less stressful and injurious to the released fish, but also, it allowed them to have “cleaner”- one species catches, which are reflected in higher revenues at the auction. There are obviously other factors not taken into account in this work, such as fishing duration, catch size, and environmental variables like water temperature or sea conditions, all important variables that may modulate survival as they significantly affect physiological, physical and behavior of escapees [20, 27–28]. The unclear mechanism and complexity involved in studying stressors and factors that cause unaccounted mortality in fish released from fishing gears, are best addressed through a combination of field and laboratory experiments [9]. This study achieved this by using realistic fishing conditions, followed by monitoring of survival, physiology and physical parameters in captivity in a more controlled environment. Modifications to commercial fishing practices such as the one proposed, should be adopted and implemented in purse seiners operating off mainland Portugal and in other European purse seine fisheries with similar bycatch/slipping issues. Fortunately this industry is known to be proactive in collaborating with the scientific community and in providing suggestions for strategies to reduce discarding and bycatch [56]. Proper uptake of the proposed modified slipping method depends on having a clear code of good practice (CoP) that should be properly disseminated, adopted and implemented.

## Supporting information

S1 Fig Survival estimates for sardine using Kaplan-Meier survival curves (dashed line), overlaid with the predictions from the selected mixture-distribution survival model M3 (solid lines with 95% confidence bands) for the three treatment replicates (1–3): Control, standard slipping (SS) and modified slipping (MS). (TIF)
