# Supplementary figures and images for: Effects of different slipping methods on the mortality of sardine, Sardina pilchardus, after purse-seine capture off the Portuguese Southern coast (Algarve)
Source: PLoS One. 2018 May 31;13(5):e0195433. doi: 10.1371/journal.pone.0195433 (PMC5978792; doi:10.1371/journal.pone.0195433)

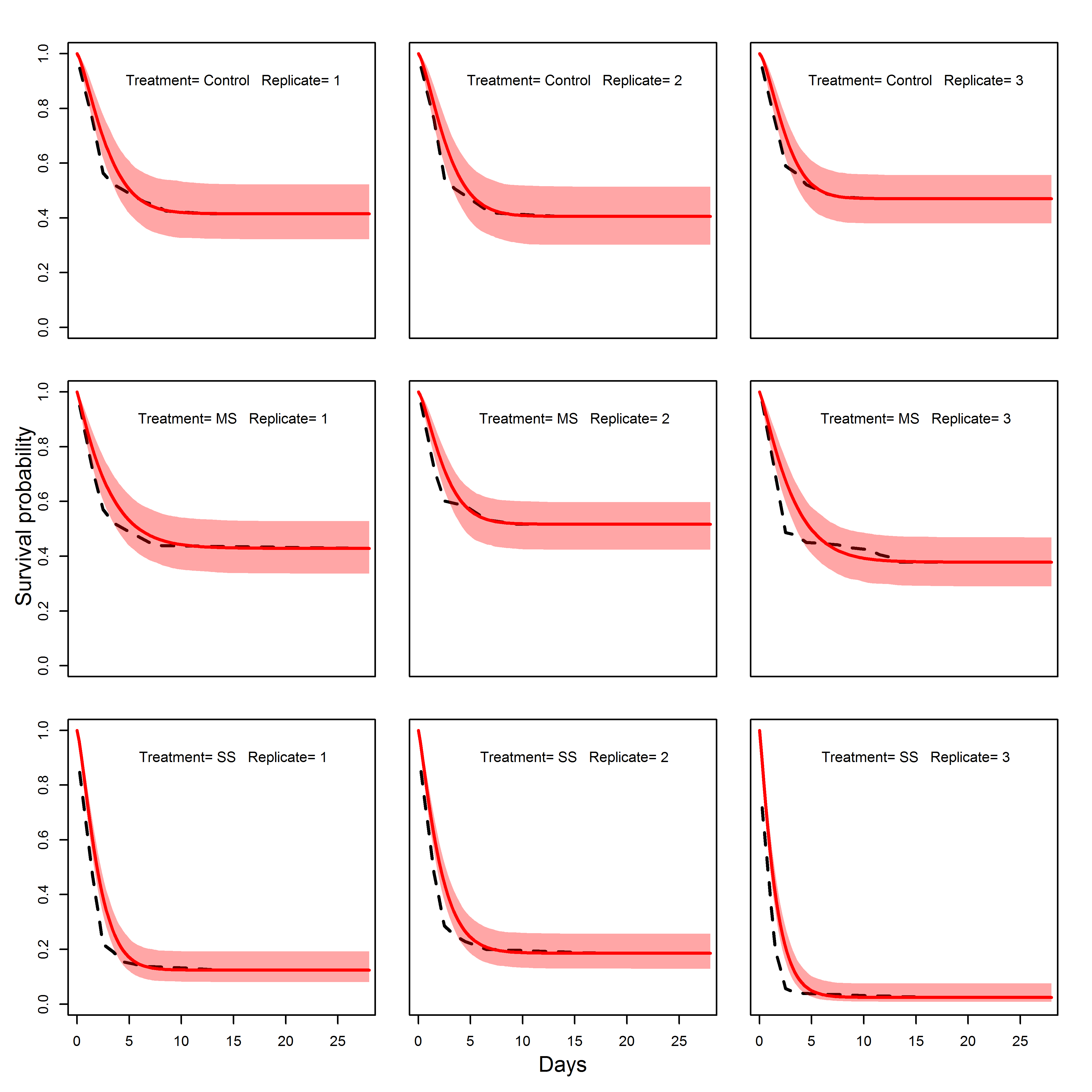

Supplement: S1 Fig — (TIF) [file pone.0195433.s001.tif]
